# Unraveling the Mystery About the Negative Valence Bias: Does Arousal Account for Processing Differences in Unpleasant Words?

**DOI:** 10.3389/fpsyg.2021.748726

**Published:** 2021-11-02

**Authors:** Lucía Vieitez, Juan Haro, Pilar Ferré, Isabel Padrón, Isabel Fraga

**Affiliations:** ^1^Cognitive Processes and Behavior Research Group, Department of Social Psychology, Basic Psychology, and Methodology, University of Santiago de Compostela, Santiago de Compostela, Spain; ^2^Department of Psychology and CRAMC, Universitat Rovira i Virgili, Tarragona, Spain

**Keywords:** arousal, valence, lexical decision task, visual word recognition, event-related potentials (ERPs)

## Abstract

Many studies have found that the emotional content of words affects visual word recognition. However, most of them have only considered affective valence, finding inconsistencies regarding the direction of the effects, especially in unpleasant words. Recent studies suggest that arousal might explain why not all unpleasant words elicit the same behavior. The aim of the present research was to study the role of arousal in unpleasant word recognition. To do that, we carried out an ERP experiment in which participants performed a lexical decision task that included unpleasant words which could vary across three levels of arousal (intermediate, high, and very high) and words which were neutral in valence and had an intermediate level of arousal. Results showed that, within unpleasant words, those intermediate in arousal evoked smaller LPC amplitudes than words that were high or very high in arousal, indicating that arousal affects unpleasant word recognition. Critically, arousal determined whether the effect of negative valence was found or not. When arousal was not matched between unpleasant and neutral valenced words, the effect of emotionality was weak in the behavioral data and absent in the ERP data. However, when arousal was intermediate in both unpleasant and neutral valenced words, larger EPN amplitudes were reported for the former, pointing to an early allocation of attention. Interestingly, these unpleasant words which had an intermediate level of arousal showed a subsequent inhibitory effect in that they evoked smaller LPC amplitudes and led to slower reaction times and more errors than neutral words. Our results highlight the relevance that the arousal level has for the study of negative valence effects in word recognition.

## Introduction

Certain stimuli appear to capture our attention more than others, and this salience is known to be determined by several factors, such as emotional content (Schacht and Sommer, 2009b). The effect of emotional content has been studied across different stimuli, as images (e.g., Cuthbert et al., 2000), films (e.g., Bos et al., 2013), and sounds (e.g., Baumgartner et al., 2006). Likewise, emotionality has been found to play a role while processing verbal stimuli such as isolated words (e.g., Kissler and Herbert, 2013), which are the focus of the present research.

Many theories have been developed trying to understand and classify emotional stimuli and emotional responses. Based on Osgood et al.’s view^1^ Osgood et al. (1957), Bradley and Lang (1999) proposed a dimensional perspective of emotion, through which emotions can be described in terms of two main dimensions: emotional valence and arousal. Emotional valence refers to the degree a stimulus is perceived as pleasant or unpleasant. On the other hand, the arousal dimension refers to the activation associated to a given stimulus, ranging between relaxing or low arousing and activating or highly arousing. These authors proposed a third dimension, dominance (that varies between under control and out of control), although this dimension is not usually manipulated in experiments due to its lack of consistency and high dependence on both emotional valence and arousal (Redondo et al., 2007). The relationship between valence and arousal has been found to be quadratic (Bradley and Lang, 1999; Redondo et al., 2007; Kousta et al., 2009; Guasch et al., 2016), meaning that the more pleasant or unpleasant a word is, the more arousing it is as well. On the contrary, words neutral in valence tend to be intermediate in arousal. This phenomenon leads to the typical boomerang-shaped graph that systematically emerges when these variables are studied (e.g., Bradley and Lang, 1999; Redondo et al., 2007; Guasch et al., 2016). Furthermore, the relation between valence and arousal is stronger in unpleasant words than in pleasant ones (Kuperman et al., 2014; Guasch et al., 2016), as pleasant words tend to have more variability in their level of arousal.

These two dimensions have been the subject of study of several investigations in the last years, and the effects of emotionality in word recognition have been studied mainly by recording the participants’ response while reading emotional and neutral words. While the specificities of the task used varied depending on the study, most of them required the participants to just read the words (silent reading) or to perform a lexical decision task (LDT; i.e., answering if the stimulus presented is a real word in the given language or not^2^). There is abundant literature showing that the emotional connotation of words affects participants’ performance (e.g., Siakaluk et al., 2016) as well as neural responses (e.g., Recio et al., 2014; see Hinojosa et al., 2019, for a review) during the LDT. For this reason, this task has been considered adequate to study the effects of word emotionality, and hence extensively used in previous research.

Regarding valence, the general effect found is that valenced words (either pleasant or unpleasant) are recognized faster than neutral ones (e.g., Kanske and Kotz, 2007; Schacht and Sommer, 2009b). Nonetheless, while pleasant words are consistently found to facilitate cognitive processing (see however, Bayer et al., 2012; Padrón et al., 2017b, for null results), unpleasant words have also been found to yield an inhibitory effect, meaning longer response latencies (Bayer et al., 2012; Padrón et al., 2017a). Both these facilitatory and inhibitory effects of negative valence have been reported by studies that analyzed lexical decision latencies for large corpora of words as well (see Kousta et al., 2009; Vinson et al., 2014; for facilitatory effects of negative valence; Larsen et al., 2008; Estes and Adelman, 2008; Kuperman et al., 2014; for inhibitory effects of negative valence). Some authors have tried to explain this “negative valence bias” (i.e., the inhibitory effect observed with unpleasant words). Concretely, Pratto and John (1991) proposed that humans possess a mechanism that allows for a rapid focalization of attention in unpleasant stimuli. This is known as the automatic vigilance hypothesis. According to Vogt et al. (2008), this preference for assigning attentional resources to unpleasant stimuli rather than to neutral and pleasant ones may be explained by the importance that unpleasant stimuli can have as a potential threat to the organism. These authors link the slowing down in the reaction times (RTs) to an instinctive “freezing” response elicited by dangerous stimuli, common in many animals. However, Estes and Verges (2008) defend that this inhibitory effect would be better explained by the increased difficulty that unpleasant words entail to disengage attention from them. Thus, when emotionality is not a relevant variable for the task, negative valence would be detrimental to performance. In fact, these authors found that the same set of unpleasant words elicited slower responses than pleasant words in a LDT but faster valence judgments.

All in all, the results regarding unpleasant word processing are inconsistent. As can be seen from the above, studies have found either a facilitatory effect, an inhibitory effect (the negative valence bias) or no effects of negative valence at all (for null effects of unpleasantness, see Larsen et al., 2006; Hinojosa et al., 2010; Scott et al., 2014). Hinojosa et al. (2019) argue that this inconsistency regarding the valence effect might be explained by differences in the arousal values of the words used in the different studies. Indeed, some studies have found different effects of unpleasant words depending on their level of arousal. Robinson et al. (2004) designed a 2 (valence) × 2 (arousal) experiment with pleasant and unpleasant words, and low and high arousal words. Unpleasant words were recognized faster when they were also high in arousal, compared to low arousal unpleasant words. The opposite happened with pleasant words, since low arousal facilitated performance compared to high arousal. These authors proposed that valence and arousal affect word recognition in an interactive way depending on the implicit tendencies that they elicit by nature. High arousal and unpleasantness would trigger an implicit avoidance tendency, while low arousal and pleasantness would elicit an approaching response. As a result, congruent conditions, as the combination of high arousal and negative valence (avoidance tendency + avoidance tendency) would facilitate word processing. These results have been replicated in other studies that used the same manipulation of valence and arousal (Citron et al., 2014a,b). Therefore, although the evidence supporting an effect of arousal by itself is not consistent (Kuperman et al., 2014), there is some evidence pointing toward the importance of this variable when valence effects are studied.

In recent years, emotionality effects have been studied using the Event-Related Potentials (ERPs) technique. In contrast with “late” measures such as RTs and errors that involve post-lexical processes, on-line measures such as ERPs are key to better provide “fine-grained” information regarding the temporal localization of emotionality effects (Kissler and Herbert, 2013). In fact, several ERP components have been reported in response to the emotional content of words, two of them being consistently found in most studies: the Early Posterior Negativity (EPN) and the Late Positive Complex (LPC).

EPN is usually reported starting approximately 200 ms after stimulus onset and it presents an occipito-temporal scalp distribution (Kissler and Herbert, 2013; Palazova, 2014). Increased amplitudes in EPN have been considered to reflect an early recognition of familiar and evolutionary relevant word forms. This facilitatory effect would be caused by an automatic and involuntary allocation of attention on intrinsically relevant stimuli (Herbert et al., 2008; Bayer et al., 2012; Palazova, 2014). Both valence and arousal have been found to be determining factors for eliciting EPN modulations (e.g., Schacht and Sommer, 2009a, for valence and Bayer et al., 2012, for arousal), so this component is usually linked to a general emotionality effect that integrates valence and arousal. However, there are some inconsistencies regarding the direction of negative valence effects in this component: While some studies have found a general facilitatory effect for emotional words, both pleasant and unpleasant, when compared to neutral ones (Herbert et al., 2008; Kissler et al., 2009; Schacht and Sommer, 2009b; Scott et al., 2009; Kissler and Herbert, 2013), others only report this facilitation for pleasant words (Schacht and Sommer, 2009a; Recio et al., 2014).

LPC, sometimes called Late Posterior Positivity (LPP), has been reported to start from 500 to 800 ms after stimulus onset and it presents a centro-parietal scalp distribution (Schacht and Sommer, 2009b; Bayer et al., 2010; Kissler and Herbert, 2013). LPC is associated with indexing a more controlled, explicit processing of emotion when compared to EPN, and it is also related with evaluation, decision making, and error detection (Citron, 2012). Increased amplitudes in LPC have thus been interpreted as a facilitatory effect of emotionality reflecting a sustained processing of evolutionary relevant stimuli (Herbert et al., 2008). However, while both valence and arousal have been found to elicit modulations in this component (e.g., Schacht and Sommer, 2009a, for valence; Bayer et al., 2012, for arousal), the direction of the effect of negative valence in LPC is, again, inconsistent. Several studies have reported an advantage for pleasant and unpleasant words over neutral ones (Fischler and Bradley, 2006; Schacht and Sommer, 2009b; Bayer et al., 2010), while others have found an advantage for pleasant words over neutral and unpleasant ones (Herbert et al., 2008; Kissler et al., 2009), or even an advantage for neutral words over pleasant and unpleasant ones (Hinojosa et al., 2009).

As can be seen from the above, it seems then that the inconsistencies regarding the direction of negative valence effects are not limited to behavioral data. Although most ERP studies point toward a facilitatory effect of positive valence in word recognition, the evidence for a general facilitatory effect (that is, for both positive and negative valence) seems to be less consistent. Note, though, that most of the above-mentioned studies have used valenced words with a high level of arousal while neutral words were intermediate or low in arousal (a table summarizing valence and arousal values and scales used in previous literature can be found as Supplementary Material). These results cannot then be interpreted as evidence for a genuine (or a lack of) valence effect, since both valence and arousal vary between emotional and neutral words (see for example, Kanske and Kotz, 2007; Herbert et al., 2008; Kissler et al., 2009; Scott et al., 2009). As previously suggested, and since higher levels of arousal have been associated with enhanced processing in both EPN and LPC time-windows, these differences in the arousal level between emotional and neutral words across studies may explain the inconsistencies regarding the effects of negative valence in word processing. Similar to Robinson et al.’s (2004) approach, a few studies have explored the interaction between valence and arousal in LDTs, this time not only at the behavioral level but in electrophysiological data as well (Hofmann et al., 2009; Bayer et al., 2012; Recio et al., 2014).

Hofmann et al. (2009) designed an experiment with low arousal pleasant and neutral words and unpleasant words that could be either low or high in arousal. These authors found that high arousal unpleasant words elicited faster RTs than low arousal unpleasant words. High arousal unpleasant words also showed higher amplitudes than both low arousal unpleasant and neutral words at an early time-window (N100). As regards LPC, higher amplitudes for high arousal unpleasant words were only reported when compared to neutral words with a low level of arousal. It is important to mention that, although high arousing words were actually high in arousal in this study (3.94 in a scale ranging from 1 to 5), the words included in the low arousal conditions had rather intermediate arousal values (around 3 in a scale ranging from 1 to 5).

Bayer et al. (2012) designed an experiment using pleasant, unpleasant, and neutral valenced words, half of them being low in arousal and the other half being high in arousal. These authors used a −3 to +3 scale for measuring valence and a scale ranging from 1 to 5 for measuring arousal (the mean in arousal was 3.7 for words high in arousal and 2.5 for words low in arousal). They found a negative valence effect, meaning slower RTs and more errors when responding to unpleasant words compared with neutral ones, and an interaction between valence and arousal, as low arousing unpleasant words elicited slower RTs and higher error rates than high arousing unpleasant ones. However, the ERP analysis did not show an effect of negative valence or an interaction between valence and arousal in either the EPN or LPC time-windows. They only found higher amplitudes for both pleasant words and highly arousing words separately, and the co-occurrence of valence and arousal effects was found to be limited to the LPC time-window.

Finally, Recio et al. (2014) designed an experiment using pleasant, unpleasant, and neutral valenced words with low, moderate, and high arousal. This study introduced for the first time a manipulation of arousal in three levels, in contrast with the low-high dichotomy present in previous literature. They used a scale ranging from −3 to + 3 for measuring valence and a scale ranging from 1 to 5 for measuring arousal, where words between 2.6 and 3 points were considered as “moderate” (these being similar to the “intermediate” ones used in the present study). They did not find an effect of negative valence but an overall facilitation (faster responses and higher amplitudes in EPN) for both pleasant and high arousal words separately. No effect of negative valence was found when comparing neutral and unpleasant words with moderate levels of arousal either. There was, nonetheless, an arousal effect (higher amplitudes in EPN for high arousing words and faster RTs when compared to words moderate or low in arousal) and an interaction between valence and arousal. Thus, high arousal unpleasant words speeded performance when compared to unpleasant words that were moderate or low in arousal. This interaction was also significant in the EPN time-window, high arousal unpleasant words showing higher amplitudes than unpleasant words that were moderate in arousal. Arousal affected neutral words in a similar way. However, neither the performance nor the ERP data were modulated by arousal in pleasant words.

In sum, recent data from studies that manipulated valence and arousal in single word processing provide evidence for the interaction between these two variables (see, however, Bayer et al., 2012, for null results). Both behavioral and electrophysiological results support Robinson et al.’s (2004) findings, as high arousal seems to entail an advantage in unpleasant word processing. There is also evidence supporting these interactive effects coming from fMRI studies (Citron et al., 2014a), analyses of lexical decision latencies for large corpora of words (Rodríguez-Ferreiro and Davies, 2019), and sentence processing studies (Bayer et al., 2010). All these results have important implications for the study of valence effects. First, if arousal can modulate the effect of negative valence, this variable should be controlled across negative and neutral conditions (as previously mentioned, this was not common in most studies). Second, this control should not be only limited to ensure that there are no differences in arousal between conditions, but also words with a similar level of arousal must be used. If not all unpleasant words are processed in the same way, the effect of negative valence reported when analyzing all these words on the whole would be a mix of the different effects that unpleasant words have depending on their level of arousal. Thus, these effects could vary depending on the selection of materials in each study^3^.

Altogether, the evidence provided by these studies suggest that the effects of valence and arousal are deeply intertwined and difficult to disentangle. But can the interaction between valence and arousal explain the inconsistency of the results regarding negative valence effects? Although the direction of this interaction seems clear (high arousal facilitates unpleasant word processing, while low arousal inhibits unpleasant word processing), ERP results about the topic are scarce and inconsistent. Early effects (Hofmann et al., 2009), EPN effects (Recio et al., 2014), LPC effects (Hofmann et al., 2009), and no effects of this interaction (Bayer et al., 2012) have all been reported. Furthermore, most of the studies that aimed to explore the interactive effects of valence and arousal present certain limitations regarding the control of the emotional variables. Some do not include a condition of neutral valence (Robinson et al., 2004), and therefore are not able to study the effect of negative valence on its own. The studies that include words neutral in valence, often only include words low and high in arousal (Bayer et al., 2012; Citron et al., 2014a,b). This does not allow for a comparison between neutral and unpleasant words with intermediate levels of arousal to be made. Hence, the effect of negative valence reported is a result of a comparison between neutral valenced words with both high arousal unpleasant words and low arousal unpleasant ones altogether (which may have different effects on word processing). Additionally, studies that introduced the condition of intermediate arousal to explore the effects of this interaction did not find any effect of negative valence in the behavioral or electrophysiological measures when unpleasant and neutral words both intermediate in arousal were compared (Hofmann et al., 2009; Recio et al., 2014). Hence, although these studies provide evidence for an arousal effect on unpleasant word processing, further research is needed to certainly demonstrate that arousal may account for the inconsistencies regarding the negative valence effect.

We developed this study with the purpose to give an answer to the following questions: (1) Does arousal affect unpleasant word recognition? (2) Can arousal account for the inconsistencies regarding the negative valence effect? and (3) Does negative valence have an inhibitory or a facilitatory effect? To achieve these goals, we designed a LDT experiment in Spanish that included neutral words in valence with an intermediate level of arousal [e.g., *sartén* (pan)] and unpleasant words that varied in their degree of arousal [intermediate, high, and very high arousal; e.g., *ceniza* (ash), *temblor* (tremor), and *avalancha* (avalanche), respectively].

To study arousal effects in negative valence, our study introduces three different levels of arousal within unpleasant words (intermediate, high, and very high). We expect to find an arousal effect, in that responses to high and very high arousal unpleasant words will be faster in comparison to unpleasant ones intermediate in arousal. As regards ERP data, we predict larger amplitudes for unpleasant words high and very high in arousal when compared to unpleasant words intermediate in arousal in EPN or LPC time-windows. However, although arousal has been found to elicit modulations in these two components, the evidence for an interaction between valence and arousal in each of them is scarce and inconsistent. Thus, we do not know for sure if these arousal effects will be limited to one component (either EPN or LPC) or present in both time-windows. Additionally, our study introduces for the first time a differentiation between high and very high arousal in unpleasant words. This will allow us to explore whether there are incremental differences in the effect of arousal on unpleasant word recognition, or if both high and very high levels of arousal affect unpleasant word recognition in a similar way instead.

To study the effect of negative valence in word recognition, and to further elucidate if arousal can account for the inconsistencies regarding this effect, several comparisons will be conducted. First, by comparing the neutral words and all the unpleasant ones (that is, those intermediate, high and very high in arousal altogether), we will test if there is an effect of negative valence when (a) the arousal is not matched between conditions and (b) there are words with different levels of arousal within the unpleasant words (as previously stated, this was the common manipulation of the emotional variables in most studies). Note, though, that these results cannot be interpreted as a genuine valence effect since both valence and arousal vary between the two conditions. We will then refer to this factor as Emotionality. As previously stated, we predict that high arousal will have a facilitatory effect in unpleasant word recognition, while intermediate levels of arousal will hinder recognition of unpleasant words instead. Thus, we expect that the comparison between neutral words and all the unpleasant ones (that is, those with intermediate, high, and very high levels of arousal) will result in the effect of Emotionality being weak or absent. Second, pairwise comparisons between neutral words and unpleasant words intermediate, high, and very high in arousal will be performed. This will allow us not only to further study the effect of word emotionality when arousal differs between conditions (by comparing neutral words intermediate in arousal with unpleasant words high and very high in arousal) but also to clarify if there is either a facilitatory or an inhibitory negative valence effect when arousal is controlled -and intermediate- between conditions (by comparing neutral words intermediate in arousal to unpleasant words that are intermediate in arousal as well). As for the two first comparisons, we expect to find either an absent effect of word emotionality or a facilitation for unpleasant words with high and very high arousal over neutral ones. As for the comparison between neutral and unpleasant words both with intermediate levels of arousal, we expect to find different results. Following Robinson et al.’s (2004) proposal, the combination of negative valence and intermediate arousal would be incongruent and therefore detrimental to word processing. This inhibitory effect of negative valence should be evidenced by slower RTs (as well as more errors) for unpleasant words intermediate in arousal when compared to neutral ones. Although prior evidence for an inhibitory effect of negative valence is restricted to behavioral data, we expect that our design will allow for it to show up in the ERP measures as well. Following the previous literature, this inhibitory effect should translate into smaller amplitudes for unpleasant words in EPN or LPC time-windows in comparison to neutral ones.

## Materials and Methods

### Participants

Thirty-six Spanish speakers (32 women; mean age 22.3 years, *SD* = 5.42) participated in this experiment. All had either normal or corrected-to-normal vision, no language difficulties or history of neurological disease, and 30 were right-handed. All were balanced bilinguals who speak Spanish and Catalan. Prior to the experiment, participants provided informed consent. They were paid 20€ for their participation.

### Materials

Two hundred forty Spanish words were selected using the emoFinder^4^ tool (Fraga et al., 2018), based on the databases of Stadthagen-González et al. (2017; 204 words) and Guasch et al. (2016; 36 words). From the 240 words selected, half of them were neutral in valence and the other half were unpleasant. The scale used for valence ranged from 1 (unpleasant) to 9 (pleasant) and a 1 to 9 scale was used for arousal as well (1, low arousal; 9, high arousal). The neutral valenced words were intermediate in arousal, with values in both variables varying between 4.5 and 5.5. A subset of 40 neutral words (IN) was randomly selected using the software Match (Van Casteren and Davis, 2007), with the aim of further analyzing the differences between neutral words and the different sets of unpleasant words. The other set of 120 words were unpleasant, and they were divided in three levels of arousal: 40 unpleasant words which were intermediate in arousal (IU), with arousal values that ranged from 4.5 to 5.5 and valence values that ranged from 1.0 to 3.5; 40 unpleasant and highly arousing words (HU), with arousal values ranging from 6 to 6.9 and valence values ranging from 1.0 to 3.5; and 40 unpleasant words very high in arousal (HHU), with arousal values ranging from 7 to 8 and valence values ranging from 1.0 to 3.5. The mean values for arousal and valence for each set of words are presented in Table 1.

**TABLE 1:** Mean and standard deviation values for each set of words in all controlled and manipulated variables.

**Word set**		**Valence**	**Arousal**	**N° letters**	**Freq.**	**Orto. N.**	**Famil.**	**Imageab.**	**Concr.**	**Contex. D.**	**NLD**
Unpleasant		2.86 (0.44)	6.34 (0.93)	7.18 (1.44)	3.81 (3.23)	2.05 (0.53)	4.77 (0.91)	4.43 (1.03)	4.56 (0.88)	1.84 (1.59)	0.64 (0.27)
	HHU	2.82 (0.37)	7.33 (0.27)	7.20 (1.38)	3.87 (2.77)	1.98 (0.43)	4.86 (0.85)	4.63 (0.91)	4.54 (0.74)	2.09 (1.71)	0.66 (0.26)
	HU	2.85 (0.46)	6.52 (0.28)	7.18 (1.55)	3.78 (3.30)	2.02 (0.57)	4.65 (1.04)	4.34 (0.96)	4.49 (0.87)	1.81 (1.55)	0.68 (0.21)
	IU	2.91 (0.48)	5.18 (0.26)	7.15 (1.41)	3.77 (3.66)	2.14 (0.56)	4.81 (0.83)	4.37 (1.18)	4.67 (1.02)	1.59 (1.50)	0.66 (0.24)
Neutral		5.06 (0.26)	5.09 (0.27)	7.14 (1.39)	3.78 (3.13)	2.04 (0.51)	4.86 (0.94)	4.40 (1.26)	4.66 (0.96)	1.65 (1.33)	0.65 (0.26)
	IN	5,13 (0.25)	5,10 (0.28)	7,25 (1.32)	3,86 (2.97)	1,98 (2.14)	4,88 (0.94)	4,31 (1.17)	4,66 (0.92)	1,70 (1.31)	0,74 (0.20)

*HHU, very high arousal unpleasant words; HU, high arousal unpleasant words; IU, intermediate arousal unpleasant words; IN, subset of intermediate arousal neutral words; Freq., frequency, Orto; N., orthographic neighbors; Famil., familiarity (1–7 scale); Imageab., imageability (1–7 scale); Concr., concreteness (1–7 scale); Contex. D., contextual diversity; NDL, normalized levensthein distance.*

*T*-tests and one-way ANOVAs were carried out in order to test if there was any difference in the emotional variables for each set. The comparison between the 120 neutral words with the 120 unpleasant ones revealed differences in both valence (*p* < 0.05) and arousal (*p* < 0.05) between the two sets of words. As for the pairwise comparisons, it was assured first that the IN subset did not differ in valence or arousal to the 120 neutral words (*p*_*s*_ > 0.05). These analyses revealed no differences in valence between the three sets of unpleasant words (IU, HU, and HHU; all *p*_*s*_ > 0.05) but significant differences were found between IN words and IU, HU, and HHU words (all *p*_*s*_ < 0.05). As for arousal, the analyses revealed differences in this variable between IU, HU, and HHU words (all *p*_*s*_ < 0.05) as well as between IN words and both HU and HHU words (all *p*_*s*_ < 0.05). Importantly, IN and IU words did not differ in arousal (*p* > 0.05).

Only low-frequency words were used (frequency ≤ 15), as high frequency words are usually associated with fast RTs, and frequency can interact with emotionality, even to the point of masking emotionality effects in some experiments (Padrón et al., 2017b). The following lexical and semantic variables, known to affect word recognition, were matched across conditions and word sets (all *p*_*s*_ > 0.05): number of letters, word frequency per million, orthographic neighbours^5^, familiarity, imageability, concreteness, contextual diversity^6^ and the Normalized Levensthein Distance between Spanish and Catalan (NLD)^7^. The data for these variables was obtained using the emoFinder (Fraga et al., 2018) and EsPal (Duchon et al., 2013) tools. The mean values for these variables in each set of words are presented in Table 1.

Finally, for the purposes of the LDT, 240 pseudowords were created using the Wuggy software, a pseudoword generator that allows for the generation of written polysyllabic pseudowords that obey a given language’s phonotactic constraints (Keuleers and Brysbaert, 2010).

### Design

As we did not orthogonally manipulate valence and arousal, we used a nested repeated measures design that includes the factor Emotionality (with two levels: neutral and unpleasant). Within the unpleasant level, the factor Arousal was manipulated (with three levels: intermediate, high, and very high). As indicated, a subset of 40 neutral words was selected for pairwise comparisons with the different levels of the factor Arousal, to further study the differences between neutral and unpleasant words.

### Procedure

Participants performed a LDT in a sound attenuated and dimly lit room while seated in a comfortable chair. Each trial began with an image of an eye displayed for 2,000 ms, which indicated to participants that in that moment they were allowed to blink. The image was followed by a fixation point (i.e., “ + ”) that appeared in the center of the screen for 500 ms. Then, the fixation point was replaced by a string of letters. The task required the participants to decide whether the string of letters was a Spanish word or not. They were instructed to press the “yes” labeled key of a keyboard with the right hand if the string of letters was a word and to press the “no” labeled key of the keyboard with the left hand if it was not a word. The string of letters remained on the screen until the participants’ response or timeout (after 2,000 ms). After responding, a feedback message (i.e., “ERROR” or “CORRECT”) was displayed for 750 ms. The order of the experimental trials was randomized for each participant. Prior to the experiment, a practice block consisting of 12 trials (6 words and 6 pseudowords) was presented, and there were two brief breaks during the experiment. The software used to display and record the responses was DMDX (Forster and Forster, 2003).

Once they finished the main task, participants answered a language history questionnaire, to assess that they had a native-like degree of proficiency in Spanish. The duration of each session was about 2 h.

### EEG Recording

The electroencephalogram (EEG) activity was recorded from 32 Ag/AgCl electrodes attached to an elastic cap (ActiCap, Brain Products, Gilching, Germany) that was positioned according to the 10–20 system. One electrode was placed beneath the left eye to monitor blinking and vertical eye movements (VEOG), and another at the outer canthus of the right eye to monitor horizontal eye movements (HEOG). All scalp electrodes were referenced online to the right earlobe and re-referenced off-line to the average of the right and left earlobes. Electrode impedances were kept below 5 kΩ. All EEG and EOG channels were amplified using an actiCHamp amplifier (Brain Products, Gilching, Germany).

Data was processed using BrainVision Analyzer 2 (Brain Products, Gilching, Germany). EEG was refiltered offline with a bandpass of 0.1–30 Hz 12 dB/oct. zerophase shift digital filter. Average ERPs were calculated per condition per participant from -200 to 800 ms relative to the onset of the word. A 200 ms pre-target period was used as baseline. Trials were rejected if the amplitude on any channel exceeded ± 75 μV, and if deflections on any channel exceeded ± 150 μV. Less than 5% of trials were rejected after applying such trimming procedures. Only correct response trials were included in the averages. On average, 104 trials per participant were kept for neutral words (35 for IN) and 104 for unpleasant words (34 for IU; 35 for HU, and 35 for HHU).

## Results

No participants were discarded due to a high percentage of errors in the task. However, six participants were excluded from the final data due to errors while recording the behavioral or EEG activity, and another one was excluded for having a low number of valid trials in one of the experimental conditions after data trimming. Both RTs that exceeded 2 *SD* of each participant’s mean and RTs lower than 250 ms or higher than 1,500 ms were treated like outliers and eliminated from the analyzed data (6.5% of the data). In addition, we excluded four words^8^ from the analyses due to a high percentage of errors (> 50%), so the final items were 236. Of those, 117 were neutral words (40 IN) and 119 were unpleasant words (40 IU, 39 HU and 40 HHU).

Regarding behavioral data (RTs and accuracy), the arousal effect was analyzed using repeated measures ANOVAs in which Arousal had three levels (IU, HU, and HHU). *T*-tests were performed to analyze Emotionality (comparing all the neutral words with all the unpleasant words). In addition, we conducted *t*-tests analyses to compare the subset of neutral words (IN) with each set of unpleasant ones (IU, HU, and HHU). All behavioral analyses were carried out by participants and items, and the analyzed factors were treated as within-participant factors in the former and as between-participants factors in the latter.

Event-related potential analyses were focused on the EPN and LPC components. To define EPN and LPC time-windows and scalp positions we used similar parameters as previous studies (e.g., Scott et al., 2009; Bayer et al., 2012). EPN was measured by computing mean amplitudes between 200 and 300 ms after word onset, and the analysis of this component included the data recorded by 17 electrodes (C3, T7, CP5, CP1, P3, P7, O1, OZ, O2, P4, P8, CP6, CP2, CZ, C4, and T8). This way, a 17 × 3 repeated-measures ANOVA and a 17 × 2 ANOVA were carried out for the factors Arousal and Emotionality, respectively. Three other 17 × 2 ANOVAs were performed to compare IN words with IU, HU, and HHU words. The time range for the LPC component was established between 420 and 630 ms after word onset, and the analysis of this component included the data from 10 electrodes (FC1, C3, CP1, PZ, P3, P4, CP2, CZ, C4, and FC2). This way, a 10 × 3 Repeated-Measures ANOVA was carried out for the factor Arousal, and a 10 × 2 ANOVA for the Emotionality factor. Additionally, three more 10 × 2 ANOVAs were performed to compare IN words with IU, HU, and HHU words. All ERP analyses were carried out by participants only, as it is common practice with this kind of measure. The main effect of electrode will not be discussed.

### Behavioral Results

The main effect of Arousal was found not to be significant in the RTs analysis [*F*_1_(2,56) = 2.47, *p* = 0.094; *F*_2_(2,116) = 1.28, *p* = 0.282]. However, this effect was significant in the error rates analysis [*F*_1_(2,56) = 5.50, *p* = 0.007; *F*_2_(2,116) = 3.59, *p* = 0.031]. Planned comparisons showed that participants committed more errors when answering to IU words than to HU (*p_1_* = 0.026; *p*_2_ = 0.098) and HHU words (*p_1_* = 0.023; *p*_2_ = 0.048), but no significant differences were found between HU and HHU words (*p_1_* > 0.05; *p*_2_ > 0.05).

Regarding Emotionality, our analysis showed a main effect of this factor, with faster RTs in neutral words than in unpleasant words. However, this effect was only significant in the participant analysis [*t*_1_(28) = 3.65, *p* = 0.001; *t*_2_(234) = 1.78, *p* = 0.076]. No main effects of Emotionality were found when analyzing error rates [*t*_1_(28) = 1.29, *p* = 0.207; *t*_2_(234) = 0.98, *p* = 0.329].

The comparison between IN and IU words was significant in RTs [*t*_1_(28) = 3.78, *p* < 0.001; *t*_2_(77) = 2.20, *p* = 0.031] and errors [*t*_1_(28) = 4.805, *p* < 0.001; *t*_2_(77) = 3.31, *p* = 0.001], showing that participants took longer to answer (and committed more errors) when responding to IU words than to IN words. The comparison between IN and HU words also showed significant differences between these two sets of words in RTs [*t*_1_(28) = 2.95, *p* = 0.006; *t*_2_(76) = 1.27, *p* = 0.207] that were only marginally significant in the error rates analysis [*t*_1_(28) = 1.92, *p* = 0.065; *t*_2_(76) = 1.90, *p* = 0.061]. Finally, the comparison between IN words and HHU words failed to show any significant effect in both RTs [*t*_1_(28) = 1.48, *p* = 0.150; *t*_2_(77) = 0.54, *p* = 0.591] and error rates analyses [*t*_1_(28) = 1.47, *p* = 0.154; *t*_2_(77) = 1.33, *p* = 0.188]. Behavioral data are presented in Table 2.

**TABLE 2:** Mean RT (in ms), and percentage of error rates (% Errors) per set of words (standard deviations in parentheses).

**Word set**	**Mean RTs**	**% Errors**
Unpleasant	687.88 (128.90)	4.61 (3.65)
HHU	682.47 (131.27)	3.54 (4.65)
HU	686.06 (129.68)	3.87 (4.08)
IU	695.34 (130.54)	6.48 (5.44)
Neutral	676.98 (122.25)	3.86 (3.51)
IN	674.68 (129.33)	2.30 (2.55)

*HHU, very high arousal unpleasant words; HU, high arousal unpleasant words; IU, intermediate arousal unpleasant words; IN, subset of intermediate arousal neutral words.*

### Event-Related Potential Results

#### Early Posterior Negativity

**FIGURE 1:**
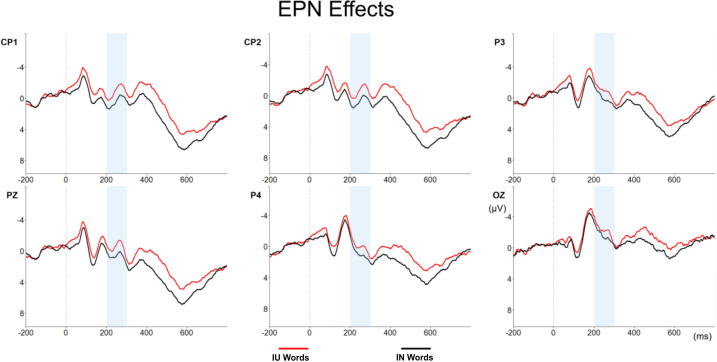
EPN effect. EPN amplitudes for the IU (red line) and the IN words (black line). The time range of the EPN component is indicated by a blue rectangle.

No effects of Arousal [*F*(2,56) = 0.96, *p* = 0.389] or Emotionality [*F*(1,28) = 2.77, *p* = 0.107] were observed in this component. However, the comparison between IN and IU words was significant [*F*(1,28) = 10.33, *p* = 0.003], IU words showing larger EPN amplitudes (−1.17 μV) than IN words (−0.29 μV) (see Figure 1). No differences were found between IN and HU words [*F*(1,28) = 3.67, *p* = 0.067] or between IN and HHU words [*F*(1,28) = 2.78, *p* = 0.107] in this time-window.

#### Late Positive Complex

**FIGURE 2:**
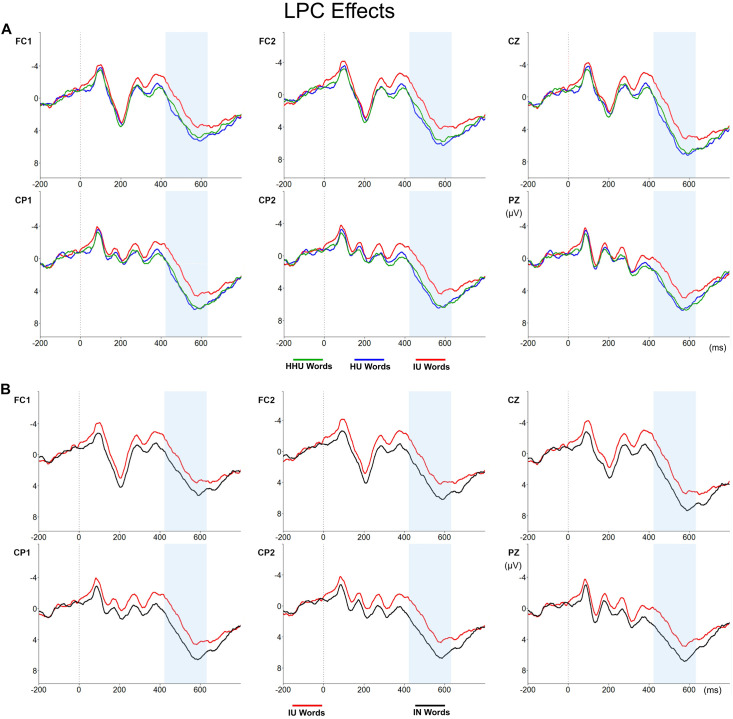
LPC effects. **(A)** LPC amplitudes for HHU (green line), HU (blue line), and IU words (red line). The time range of the LPC component is indicated by a blue rectangle. **(B)** LPC amplitudes for the IU (red line) and the IN words (black line). The time range of the LPC component is indicated by a blue rectangle.

Regarding the Arousal factor, the analysis showed a significant main effect of arousal [*F*(2,56) = 12.99, *p* < 0.001]. Both HU and HHU words elicited larger LPC amplitudes (3.56 and 3.88 μV, respectively) than IU words (1.98 μV) (*p*_*s*_ < 0.001). No differences were found between HU and HHU words (*p* > 0.05) (see Figure 2A). The ANOVA for the Emotionality factor did not show any Emotionality effect on LPC amplitudes [*F*(1,28) = 0.02, *p* = 0.893]. Finally, the comparison between IN and IU words was significant [*F*(1,28) = 48.95; *p* < 0.001], showing that IN words elicited larger LPC amplitudes (3.91 μV) when compared to IU words (1.98 μV) (see Figure 2B). However, there were not differences between either IN and HU words [*F*(1,28) = 0.01, *p* = 0.938] or IN and HHU words [*F*(1,28) = 0.86, *p* = 0.361] in the LPC time-window.

## Discussion

The central aim of this study was to explore the possible effects of arousal on unpleasant word recognition. This way, both emotional valence and arousal were manipulated in a LDT. In addition, we carried out a series of comparisons between neutral and unpleasant words with different levels of arousal, aiming to further elucidate if these arousal effects can account for the inconsistencies reported in previous literature regarding the effect of negative valence.

First, our analyses showed that arousal did affect unpleasant word recognition. While arousal effects were not significant in the EPN time-window, unpleasant words high (HU) and very high in arousal (HHU) elicited larger LPC amplitudes in comparison to unpleasant words intermediate in arousal (IU), indicating a preferential processing of the former two when compared to the latter. Regarding behavioral data, arousal effects were significant in the error rates analysis, as participants committed less errors when responding to unpleasant words very high in arousal (HHU) than to those intermediate in arousal (IU), yet no significant differences were found in RTs. Thus, it seems that not all unpleasant words were processed in the same way. These results could be interpreted in terms of an increased difficulty to process unpleasant words intermediate in arousal (IU) when compared to unpleasant ones of high (HU) or very high arousal (HHU), or, in line with Robinson et al.’s proposal and previous findings in the matter (Robinson et al., 2004; Citron et al., 2014a,b; Recio et al., 2014), as a facilitatory effect of high arousal in unpleasant word recognition.

Regarding emotionality effects, the comparison between all the neutral words and all the unpleasant words considered together did not show significant effects in any time-window in the ERP data. Emotionality did not critically affect performance either, and, although participants were faster responding to neutral words than to unpleasant ones, this effect was only significant in the participant analysis, and no differences were found between unpleasant and neutral words in error rates. In view of these results, it could seem that there are no effects of negative valence in word processing, yet these results were obtained comparing neutral and unpleasant words that differed in arousal, and therefore cannot be interpreted in terms of valence only. Interestingly, most valence effects reported in the literature correspond to a comparison between unpleasant words high in arousal and neutral words low in arousal. These studies often report a facilitation for unpleasant words over neutral ones (e.g., Kanske and Kotz, 2007; Schacht and Sommer, 2009a) that was not replicated by our data. While unpleasant words tended to be high or very high in arousal in these studies (between 3.5 and 4 in a scale from 1 to 5), neutral words tended to be low in arousal (between 1.5 and 1.8 in a scale from 1 to 5). As the neutral words used in our study had intermediate levels of arousal, the greater difference in arousal between unpleasant and neutral words in these studies in comparison to ours may account for the different results. Furthermore, as previously mentioned, this facilitatory effect of negative valence is quite inconsistent, and most of the studies that have found a facilitation for unpleasant words in comparison to neutral ones in EPN have not observed the same effects in LPC (Herbert et al., 2008; Kissler et al., 2009; Schacht and Sommer, 2009b; Kissler and Herbert, 2013). Similarly, most of the studies that have reported a facilitatory effect for unpleasant words in LPC did not find the same effect in EPN (Schacht and Sommer, 2009a) or just did not report any effects of emotionality on this component at all (Herbert et al., 2006; Kanske and Kotz, 2007). For these reasons, the absence of an effect of negative valence when arousal is not controlled between unpleasant and neutral words is not surprising.

Moreover, pairwise comparisons between the subset of neutral words (IN) and each set of unpleasant ones (IU, HU, and HHU) led to interesting results. On the one hand, both unpleasant words high (HU) and very high in arousal (HHU) seemed to be processed in a similar way to neutral ones, since none of the pairwise comparisons showed statistically significant differences between these sets of words (for HU words these differences were weak and only significant in the behavioral data, and for HHU words these differences were not significant either in the ERP or in the behavioral data). On the other hand, the comparison between unpleasant words intermediate in arousal (IU) and neutral words (IN) showed significant differences in various stages of word processing. As for EPN, IU words elicited larger amplitudes than neutral words, thus pointing toward an early allocation of attentional resources in IU words. Considering that this early effect was only significant when both unpleasant and neutral words were intermediate in arousal (but did not arise when HU and HHU words were compared to neutral ones), it seems that valence effects on EPN amplitudes were somehow related to arousal, even though no effects of arousal were significant in this time-window. One interpretation of this finding could be related to the fact that emotionality effects in EPN have been frequently associated with a general emotionality effect that does not discriminate between valence and arousal, but that is more related to the emotional relevance of the stimulus (Citron, 2012). As was previously mentioned, there is a tendency for unpleasant words to be high in arousal as well, and for neutral words to be intermediate or low in arousal. Thus, IU words would entail a combination of valence and arousal quite uncommon in natural contexts of language, or, as in Robinson et al.’s (2004) terms, an “incongruent” combination of valence and arousal. This could explain their salience over the other sets of words and hence this early focus of attention on them. Critically, differences between IN and IU words were also significant in a later time-window. IU words exhibited smaller amplitudes than neutral words in LPC, a component that has been usually associated with evaluation, decision making, and error detection (Citron, 2012). Thus, this early allocation of attention in IU words seems to have led to a detrimental processing of these unpleasant words in later stages of word processing. Consequently, IU words elicited slower and less accurate responses than neutral words in the LDT. These results would point to an inhibitory effect of negative valence, the so called “negative valence bias.” Whilst behavioral evidence for this effect was reported by other studies (e.g., Bayer et al., 2012; Padrón et al., 2017a), we present novel results regarding the inhibitory effect of negative valence in ERP data. Again, this effect seems to appear only when both neutral and unpleasant words have intermediate levels of arousal (two conditions not usually included in previous literature).

The divergences between our results on the effects of negative valence and those reported by previous studies can be related to different causes. Given that the results of this study clearly evidence that arousal affects how unpleasant words are processed, it is possible that differences in the specific manipulation of the emotional variables (and the scales used to measure them) may account for the disparate findings. Both Hofmann et al. (2009) and Recio et al. (2014) measured valence in a scale ranging from −3 to + 3 and arousal in a scale ranging from 1 to 5, while we measured both valence and arousal in a scale ranging from 1 to 9. Furthermore, words with equivalent levels of arousal are labeled as low arousing in some studies (as in Hofmann et al., 2009) and as moderate or intermediate in arousal in others (as in Recio et al., 2014). Other methodological differences, as the source of the normative ratings for the emotional variables, might as well have affected the comparison between studies and the replicability of the results. Additionally, we find necessary to point out that we did not include pleasant words in our design, but only unpleasant and neutral ones (most previous studies used pleasant, neutral, and unpleasant words). While this allowed us to focus our study on the effects of negative valence, the absence of pleasant words may have influenced the results. Adelman and Estes (2013) state that the effects of valence can be influenced by the selection of the materials. Emotional words, especially unpleasant ones, are less common than neutral words in the natural presentation of language, so experimental conditions may create a context where the proportion of unpleasant stimuli is abnormally high, and this could cause negative valence to have an unusual relevance for the participant. According to this statement, while we included a high number of neutral words, the absence of pleasant words could have affected the naturality of our stimulus list and driven participants attention to unpleasant words. This could have happened as well in the study by Hofmann et al. (2009). Out of the four sets of words used there, three of them were low in arousal and only one had high levels of arousal. Critically, these words (high arousal unpleasant words) were the ones that participants processed differently from the others.

Finally, we find interesting to point out that, while ERP data showed that HU and HHU words were processed in a similar way and no differences between these sets of words were found at the behavioral level, only HHU words significantly affected performance. Thus, although both HU and HHU words elicited higher amplitudes than IU words in LPC, the effects of arousal in behavioral data were limited to the error rates analysis and to the comparison between HHU and IU words. Our specific manipulation of the materials might explain these results, as HU and HHU words were closer in arousal than HU and IU words. Future research should explore the linearity of arousal effects by conducting regression analyses with arousal as a continuous variable.

## Conclusion

Although further research is needed to contrast our results and to explore the complex interaction between valence and arousal in the different stages of word processing, our data clearly evidence that not all unpleasant words are processed in the same way. Our results show an effect of arousal in unpleasant word recognition, so that unpleasant words intermediate in arousal evoked smaller LPC amplitudes than unpleasant words that were high or very high in arousal, this probably explaining the absence of an emotionality effect when all of them were compared together with neutral ones. Critically, arousal determined whether an effect of negative valence was found or not. Unpleasant words were only processed differently from neutral ones, both in EPN and LPC, when they were intermediate in arousal, proving that arousal can indeed account for previous inconsistencies regarding negative valence effects. This new evidence strongly supports the fact that both valence and arousal must be considered when studying the effect of emotional connotation in language processing.

## Data Availability Statement

The raw data supporting the conclusions of this article will be made available by the authors, without undue reservation.

## Ethics Statement

The studies involving human participants were reviewed and approved by Comitè Ètic d’Investigació en Persones, Societat i MediAmbient (CEIPSA-2021-PR-0015). The patients/participants provided their written informed consent to participate in this study.

## Author Contributions

LV, PF, and IF contributed to the conception and design of the study. LV selected the materials and wrote the first draft of the manuscript. JH programmed the experiment. LV and JH carried out the statistical analysis. IP contributed to data presentation and visualization. All authors contributed to manuscript revision, read, and approved the submitted version.

## Conflict of Interest

The authors declare that the research was conducted in the absence of any commercial or financial relationships that could be construed as a potential conflict of interest.

## Publisher’s Note

All claims expressed in this article are solely those of the authors and do not necessarily represent those of their affiliated organizations, or those of the publisher, the editors and the reviewers. Any product that may be evaluated in this article, or claim that may be made by its manufacturer, is not guaranteed or endorsed by the publisher.

## Footnotes

1 Osgood’s semantic differential scales identify three dimensions that can measure people’s attitudes toward nearly anything. These three dimensions were: evaluation (e.g., good/bad); potency or power (e.g., strong/weak); and activation or movement (e.g., fast/slow).

2 For example, “*aguacero*” (downpour) is a real word in Spanish, while “*avazgero*” is not. These “fake words” are commonly denominated pseudowords, and while they mimic the phonological and orthographic structure of a real word, they do not actually exist in the target language.

3 For example, a mean of 5 can be obtained both with words intermediate in arousal or a compound of words with low and high arousal. In the first case, all unpleasant words would be yielding the same effect, however, in the second case the effects of low and high arousal unpleasant words will be counterbalancing each other. Additionally, the same mean can be obtained with words low, intermediate, and high in arousal, or even with many words sightly low in arousal and a few with high levels of arousal. This way, the specific selection of the materials of each study could determine the valence effect obtained.

4 EmoFinder (Fraga et al., 2018) is a web-based search engine for Spanish word properties taken from different normative databases. It includes the normative ratings in emotional dimensions (e.g., valence and arousal) and discrete emotional categories (fear, disgust, anger, happiness, and sadness) for 16,375 different words. It also provides values for lexical properties as familiarity, imageability or concreteness.

5 Indicated by the Levenshtein Distance, neighborhood refers to the level of similarity in terms of number of deletions, insertions, or substitutions required to transform one word into another. The metric is calculated as a mean of the 20 nearest neighbors.

6 The term “contextual diversity” refers to the number of contexts in which a word appears.

7 As participants were Spanish-Catalan bilinguals, we decided to control the orthographic similarity of the words used in both languages, as it is known that this variable affects word processing (Comesaña et al., 2013). With this purpose, we calculated the Normalized Levenshtein Distance, a measure of this orthographic resemblance between the languages, using the online tool NIM (Guasch et al., 2013).

8 The words excluded were *cinc* [zinc] (neutral), *galeón* [galleon] (neutral), *surco* [groove] (neutral), and *hedor* [stink] (HU).
