# Supplementary material for: Unraveling the Mystery About the Negative Valence Bias: Does Arousal Account for Processing Differences in Unpleasant Words?
Source: Front Psychol. 2021 Nov 2;12:748726. doi: 10.3389/fpsyg.2021.748726 (PMC8593103; doi:10.3389/fpsyg.2021.748726)
Supplement: Supplementary file 1 [file Table_1.pdf]

Supplementary Table 1. Task, mean values of valence and arousal, scales used, and source of the ratings for previous literature regarding emotionality effects on single-word processing.

Study	Task	Mean values for valence and arousal	Scales for valence and arousal	Source of the ratings
Herbert et al. (2006)	Covert evaluation (in valence)	60 U: V = 2.9; A = 5.5<br>60 N: V = 4.7; A = 3.8<br>60 P: V = 6.6; A = 5.3	V: 1-9<br>A: 1-9	A sample of 45 students
Kissler et al. (2007)	Silent Reading	60 U: V = 1.9; A = 5.8<br>60 N: V = 5.1; A = 2.2<br>60 P: V = 7.4; A = 5.7	V: 1-9<br>A: 1-9	A sample of 45 students
Kanske & Kotz (2007)	LDT and go/no go LDT	60 U: V = 1.3; A = 3.5<br>120 N: V = 3.1; A = 1.5<br>60 P: V = 4.6; A = 3.2	V: 1-5<br>A: 1-5	A sample of 40 students for valence and 30 for arousal
Estes & Vergers (2008)	LDT and valence judgements	20 U: V = 2.1; A = 2.8<br>20 P: V = 3.6; A = 2.4	V: 1-5<br>A: 1-5	A sample of 35 students
Herbert et al. (2008)	Silent Reading	60 U: V = 2.7; A = 5.7<br>60 N: V = 5.0; A = 3.4<br>60 P: V = 6.6; A = 5.5	V: 1-9<br>A: 1-9	A sample of 45 students
Kissler et al. (2009)	Silent Reading	66 U: V = 2.6; A = 5.6<br>66 N: V = 5.1; A = 3.1<br>66 P: V = 7.0; A = 5.4	V: 1-9<br>A: 1-9	A sample of 45 students
Kousta et al. (2009)	LDT	40 U: V = 2.5; A = 5.6<br>40 N: V = 5.0; A = 4.4<br>40 P: V = 7.5; A = 5.8	V: 1-9<br>A: 1-9	Their own dataset
Hoffmann et al. (2009)	LDT	50 LP: V = 1.6; A = 3.2<br>50 LN: V = 0; A = 3.1<br>50 LU: V = -1.6; A = 3.2<br>50 HU: V = -1.6; A = 3.9	V: -3 to +3<br>A: 1-5	The Berlin Affective Word List (BAWL)
Scott et al. (2009)	LDT	80 U: V = 2.5; A = 6.6<br>80 N: V = 5.2; A = 4.4<br>80 P: V = 7.7; A = 6.6	V: 1-9<br>A: 1-9	The Affective Norms for English Words database (ANEW)
Schacht & Sommer (2009a)	LDT	40 U: V = -2.2; A = 4.0<br>40 N: V = 0.1; A = 1.8<br>40 P: V = 2.0; A = 3.1	V: -3 to +3<br>A: 1-5	Their own datasets

Study	Task	Mean values for valence and arousal	Scales for valence and arousal	Source of the ratings
Schacht & Sommer (2009b) Exp. 1	LDT	80 U: V = -2.2; A = 4.0<br>80 N: V = 0.1; A = 1.8<br>80 P: V = 2.0; A = 3.2	V: -3 to +3<br>A: 1-5	Their own datasets
Palazova et al. (2011)	LDT	60 U: V = -1.8; A = 3.8<br>60 N: V = 0.1; A = 2.5<br>60 P: V = 2.0; A = 2.5	V: -3 to +3<br>A: 1-5	Their own dataset and the BAWL
Bayer et al. (2012)	LDT	30 LU: V = -1.3; A = 2.5<br>30 HU: V = -1.3; A = 3.7<br>30 LN: V = 0.1 A = 2.5<br>30 HN: V = 0.1; A = 3.7<br>30 LP: V = 1.5; A = 2.5<br>30 HP: V = 1.5; A = 3.6	V: -3 to +3<br>A: 1-5	The BAWL
Kissler & Herbert (2013)	Silent reading	46 U: V = 2.0; A = 5.7<br>46 N: V = 5.2; A = 2.2<br>46 P: V = 7.5; A = 5.8	V: 1-9<br>A: 1-9	A sample of 45 students
Citron et al. (2014a)	LDT	35 LU: V = -1.3; A = 3.5<br>35 HU: V = -1.8; A = 4.6<br>35 N: V = 0.2; A = 3.3<br>35 LP: V = 1.5; A = 3.4<br>35 HP: V = 1.9; A = 4.5	V: -3 to +3<br>A: 1-7	Their own dataset
Citron et al. (2014b)	LDT	25 LU: V = -1.4; A = 3.6<br>25 HU: V = -1.6; A = 4.6<br>50 N: V = 0.2; A = 2.5<br>25 LP: V = 1.6; A = 3.6<br>25 HP: V = 1.9; A = 4.5	V: -3 to +3<br>A: 1-7	The Sussex Affective Word List (SAWL)
Recio et al. (2014)	LDT	53 LU: V = -1.4; A = 2.2<br>53 MU: V = -1.4; A = 2.8<br>53 HU: V = -1.6; A = 3.6<br>53 LN: V = 0; A = 2.2<br>53 MN: V = 0.1; A = 2.8<br>53 HN: V = 0; A = 3.5<br>53 LP: V = 1.5; A = 2.1<br>53 MP: V = 1.6; A = 2.8<br>53 HP: V = 1.7; A = 3.5	V: -3 to +3<br>A: 1-5	The BAWL

Study	Task	Mean values for valence and arousal	Scales for valence and arousal	Source of the ratings
Schindler & Kissler (2016)	Valence judgements and passive reading	70 U: V = 2.9; A = 5.6<br>70 N: V = 5.1; A = 2.6<br>70 P: V = 7.1; A = 5.5	V: 1-9<br>A: 1-9	Ratings from their own dataset

U, Unpleasant; N, Neutral; P, Pleasant; V, Valence; A, Arousal; LU, Low arousal unpleasant words; MU, Moderate arousal unpleasant words; HU, High arousal unpleasant words; LN, Low arousal neutral words; MN, Moderate arousal neutral words; HN, High arousal neutral words; LP, Low arousal pleasant words; MP, Moderate arousal pleasant words; HP, High arousal pleasant words.
